# Virtually coupled resonators with modal dominance for improved sensitivity and bandwidth

**DOI:** 10.1038/s41378-025-00897-4

**Published:** 2025-04-03

**Authors:** Zhao Zhang, Han Li, Cheng Hou, Yongcun Hao, Hemin Zhang, Honglong Chang

**Affiliations:** Ministry of Education Key Laboratory of Micro and Nano Systems for Aerospace, School of Mechanical Engineering, Northwestern Polytechnical University, 710072 Xi’an, China

**Keywords:** Engineering, Electrical and electronic engineering

## Abstract

Mode-localized sensors have attracted significant attention due to their exceptional sensitivity and inherent ability to reject common-mode noise. This high sensitivity arises from the substantial shifts in resonator amplitudes induced by energy confinement in weakly coupled resonators. Despite their promising attributes, there has been limited research on the mechanisms of energy confinement. This paper presents both qualitative and quantitative analyses of energy confinement within weakly coupled resonators and concludes them as the concept of modal dominance. This concept elucidates that mode frequencies are predominantly dictated by the natural frequencies of the internal resonators, facilitating spatial energy confinement. Based on this modal dominance, a novel concept of virtually coupled resonators is proposed, which obviates the need for physical coupling structures. Instead, energy confinement is achieved through a frequency offset between two independent resonators, resulting in a similar amplitude ratio output and enhanced sensitivity. To further enhance performance, a double-closed-loop control scheme is developed for virtually coupled resonators, expanding the bandwidth in comparison to weakly coupled resonators. Experimental results validate the feasibility of virtually coupled resonators and the double-closed-loop control, demonstrating a 2.7-fold improvement in amplitude ratio sensitivity and at least a four-fold enhancement in bandwidth relative to weakly coupled resonators with identical parameters.

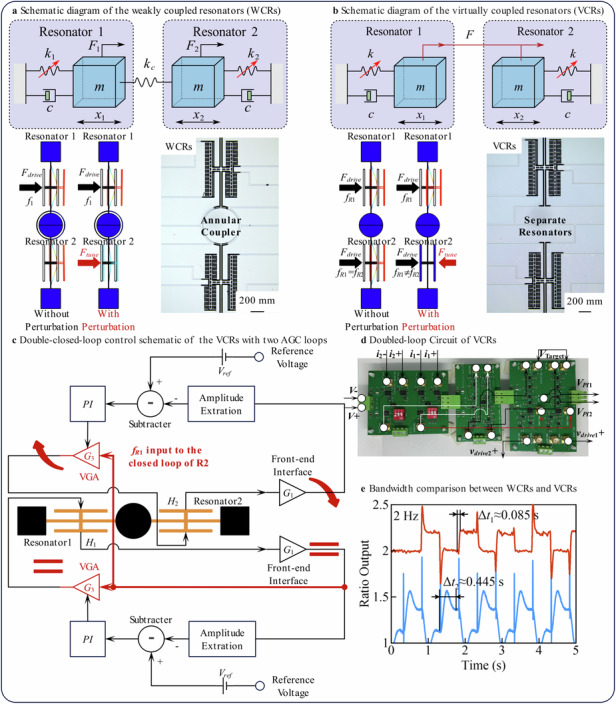

## Introduction

In recent years, significant advancements in theories and sensor technologies focusing on energy flow in coupled resonators have emerged^1–4^. Among these developments, mode-localized sensors, which leverage the mode localization phenomenon, have gained prominence due to their exceptional parametric sensitivity and superior rejection of common-mode noise, outperforming traditional frequency output resonators^5–12^. The concept of mode localization, first introduced in the 1980s by C.H. Hodges and J. Woodhouse through their pioneering work on Anderson localization^13^ in structural dynamics, describes how energy, when disrupted by external perturbations, remains confined close to its source rather than propagating indefinitely^14–16^. This energy confinement is the theoretical foundation of mode-localized sensors. It changes the amplitudes of resonators dramatically and enlarges the measured signal as the amplitude ratio (AR) of the resonators significantly^5,17–20^. Therefore, various physical quantities, such as mass^21^, acceleration^22^, force^23^, voltage^19^, current^24^, etc.^25,26^ have been measured using this principle.

Hodges’s theory posits that energy confinement in mode-localized sensors is predominantly due to the disorder induced in symmetric weakly coupled resonators (WCRs) by external perturbations^5^. However, this explanation cannot fully reflect how severely the energy is confined to a specific mode or resonator under varying conditions. This gap in understanding has not only limited the effective exploitation of this phenomenon but also impeded further enhancements in the performance of mode-localized sensors.

This paper addresses the gap in understanding energy confinement in WCRs. Specifically, it investigates the modal dominance of the WCRs, where the mode frequencies are primarily dictated by the natural frequencies of the internal resonators. The frequency offsets between the mode and natural frequencies define the resonant states of the resonators within the WCRs, leading to spatial energy confinement. Building on this foundational insight, the novel concept of virtually coupled resonators (VCRs) is introduced in this study. Derived from the modal dominance observed in WCRs, VCRs eliminate the need for physical coupling structures and instead couple two separate resonators via the frequencies of the driving force. By deliberately setting the frequency offset between two discrete resonators, VCRs can achieve similar energy confinement and AR output with WCRs. Moreover, the distinct structural features of VCRs facilitate a novel double-closed-loop control scheme that enhances both sensitivity and bandwidth performance compared to traditional WCRs. Experimental results validate the proposed concepts, demonstrating significant improvements in AR sensitivity and bandwidth compared to WCRs.

## Discussion

### Modal dominance in weakly coupled resonators

**Fig. 1:**
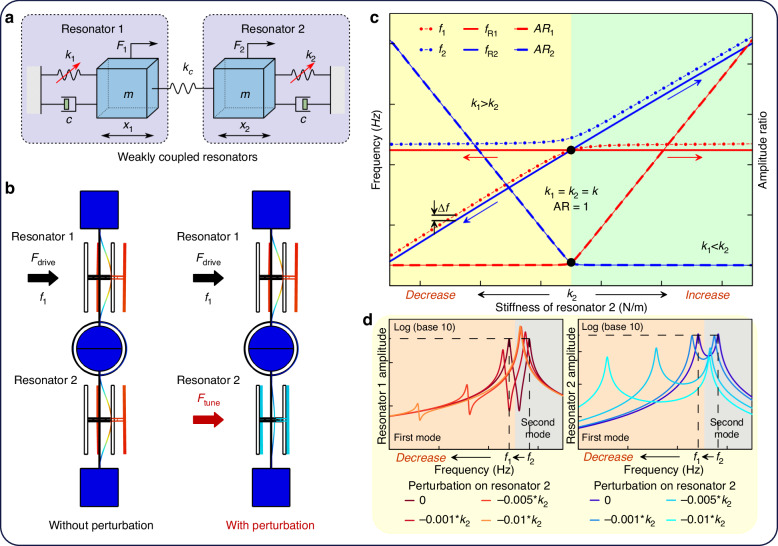
Models of WCRs and their amplitude/AR variations. **a** Mass–stiffness–damper model of WCRs. **b** COMSOL simulation of WCRs with and without perturbation. **c** Mode frequencies, AR of WCRs, and natural frequencies of the two resonators. **d** Amplitude–frequency responses of Resonator 1 and Resonator 2 under negative–stiffness perturbations applied to Resonator 2

WCRs are the core of a mode-localized sensor and typically simplified to a mass–stiffness–damper model, as shown in Fig. 1a. Each resonator is characterized by its mass (*m*), stiffness (*k*_1_ and *k*_2_ for the two resonators, and *k*_*c*_ for the coupling structure) and damping coefficient (*c*). *F*_*i*_ represents the driving force applied to the *i*-th resonator (*i* = 1, 2), and *x*_*i*_ denotes the displacement of the *i*-th resonator. In WCRs, the coupling stiffness *k*_*c*_ is much smaller than the stiffness of the individual resonators *k*_*i*_ (typically *k*_*c*_/*k*_*i*_ ≈ 0.001), with their ratio defined as the coupling coefficient *κ* = *k*_*c*_/*k*_*i*_^22^. The corresponding equations of motion in Fig. 1a are given by:

1 $$\left\{\begin{array}{c}m{\ddot{x}}_{1}+c{\dot{x}}_{1}+\left({k}_{1}+{k}_{c}\right){x}_{1}-{k}_{c}{x}_{2}={F}_{1},\\ m{\ddot{x}}_{2}+c{\dot{x}}_{2}+\left({k}_{2}+{k}_{c}\right){x}_{2}-{k}_{c}{x}_{1}={F}_{2}.\end{array}\right.$$

Stiffness perturbations from measurands can impact the stiffness and, consequently, the amplitudes of the resonators in the WCRs. The COMSOL simulations shown in Fig. 1b vividly illustrate the variation in amplitudes of the WCRs, both with and without perturbations. When Resonator 1 is driven (*F*_1_ = *F*_*drive*_, *F*_2_ = 0) and no stiffness perturbations are present, both resonators in the WCRs resonate with identical amplitudes. However, when the perturbations alter the stiffness of Resonator 2, the amplitude of Resonator 1 increases, while that of Resonator 2 decreases. The mechanical amplification mechanism, resulting from their AR output, is crucial for the high parametric sensitivity of mode-localized sensors. The AR_*i-WCRs*_ in the two modes and the sensitivity of the *S*_AR*-WCRs*_ can be expressed as follows:

2 $$\left\{ \begin{array}{ll}{\mathrm{AR}}_{i-WCRs}&=\frac{X_1\left( \omega _i \right)}{X_2\left( \omega _i \right)}=\frac{-m\omega _{i}^{2}+jc\omega _i+k_2+k_c}{k_c},i=1,2,\\\left| S_{\mathrm{AR}-WCRs} \right|&=\frac{1}{k_c}=\frac{1}{\kappa k}.\end{array} \right.$$

where *X*_*i*_(*ω*_*i*_) represents the amplitudes of the resonators at mode frequencies; and *ω*_*i*_ denotes the mode angular frequencies of the WCRs. The AR_*i-WCRs*_ are plotted against the stiffness of Resonator 2 in Fig. 1c. The amplitude–frequency responses of Resonator 1 and Resonator 2 are shown in Fig. 1d.

From Eq. (2), it can be observed that the AR sensitivity of the WCRs is primarily dependent on its coupling stiffness. However, the sensitivity is also constrained by the condition to avoid mode aliasing, which requires *κQ* > 1 (*Q* represents the quality factor of the resonators). Therefore, if the *Q* of the WCRs cannot be simultaneously improved, its AR sensitivity cannot be indefinitely enhanced by merely reducing the coupling stiffness.

#### Frequencies offsets of the weakly coupled resonators

The mode frequencies (*f*_*i*_ = *ω*_*i*_/2*π*, *i* = 1, 2) for the two resonant modes of the WCRs are derived as follows^27,28^. To ensure consistency, the mode frequencies are ordered by magnitude, with the smaller frequency designated as the first mode (*f*_1_) and the larger frequency as the second mode (*f*_2_). The mode frequency curves as a function of the stiffness of Resonator 2 are shown in Fig. 1c. The black point represents the condition where the stiffness of both resonators is equal (*k*_1_ = *k*_2_ = *k*), corresponding to the minimum frequency splitting between the two modes.

3 $$\left\{\begin{array}{c}{f}_{1}=\frac{{\omega }_{1}}{2\pi }=\frac{1}{2\pi }\sqrt{\frac{{k}_{1}+{k}_{2}+2{k}_{c}-\sqrt{{\left({k}_{1}-{k}_{2}\right)}^{2}+4{k}_{c}^{2}}}{2m}},\\ {f}_{2}=\frac{{\omega }_{2}}{2\pi }=\frac{1}{2\pi }\sqrt{\frac{{k}_{1}+{k}_{2}+2{k}_{c}+\sqrt{{\left({k}_{1}-{k}_{2}\right)}^{2}+4{k}_{c}^{2}}}{2m}}.\end{array}\right.$$

When a negative stiffness perturbation is applied to Resonator 2, the stiffness of Resonator 1 exceeds that of Resonator 2 (*k*_1_ > *k*_2_), as indicated by the yellow region in Fig. 1c. Under these conditions, *f*_1_ decreases significantly, while *f*_2_ exhibits only a slight reduction. Conversely, applying a positive stiffness perturbation to Resonator 2 reverses the trend in mode frequencies variations.

Assuming the ratio of coupling stiffness to the resonator stiffness approaches zero, i.e., *lim*(*k*_*c*_/*k*_*i*_ → 0)^14,29,30^, the coupling structure effectively only connects the internal resonators without significantly affecting their stiffness. Under this assumption, the equations can be approximated from Eq. (1) by neglecting the terms involving *k*_*c*_/*k*_*i*_:

4 $$\left\{\begin{array}{c}m{\ddot{x}}_{1}+c{\dot{x}}_{1}+{k}_{1}{x}_{1}={F}_{1},\\ m{\ddot{x}}_{2}+c{\dot{x}}_{2}+{k}_{2}{x}_{2}={F}_{2}.\end{array}\right.$$

This assumption effectively treats the coupling structure in the WCRs as negligible, leaving the two resonators as independent. The natural frequencies (*f*_*Ri*_) of these resonators can then be calculated as^27,28^:

5 $$\left\{\begin{array}{c}{f}_{R1}=\frac{{\omega }_{R1}}{2\pi }=\frac{1}{2\pi }\sqrt{\frac{{k}_{1}}{m}},\\ {f}_{R2}=\frac{{\omega }_{R2}}{2\pi }=\frac{1}{2\pi }\sqrt{\frac{{k}_{2}}{m}}.\end{array}\right.$$

The curves of natural frequencies versus the stiffness of Resonator 2 are shown in Fig. 1c. At the black point, the mode frequencies of the WCRs are nearly identical to the natural frequencies of the resonators. When the stiffness of Resonator 1 exceeds that of Resonator 2 (*k*_1_ > *k*_2_), *f*_1_ is almost equal to *f*_*R*2_, and *f*_2_ is nearly equal to *f*_*R*1_. The slight discrepancy between the mode frequencies and natural frequencies, denoted as Δ*f* in Fig. 1c, results from the coupling stiffness *k*_*c*_. Conversely, when *k*_1_ < *k*_2_, the relationship between mode and natural frequencies is reversed.

6 $$\left\{\begin{array}{l}{k}_{1}={k}_{2}=k:{f}_{1}=\frac{1}{2\pi }\sqrt{\frac{k}{m}}={f}_{R1}={f}_{R2}\approx \frac{1}{2\pi }\sqrt{\frac{k+2{k}_{c}}{m}}={f}_{2},\\ {k}_{1} \,>\, {k}_{2}:\left\{\begin{array}{c}{f}_{1}=\frac{{\omega }_{1}}{2\pi }\approx \frac{1}{2\pi }\sqrt{\frac{{k}_{2}}{m}}={f}_{R2},\\ {f}_{2}=\frac{{\omega }_{2}}{2\pi }\approx \frac{1}{2\pi }\sqrt{\frac{{k}_{1}}{m}}={f}_{R1}.\end{array}\right.,\\ {k}_{1} \,<\, {k}_{2}:\left\{\begin{array}{c}{f}_{1}=\frac{{\omega }_{1}}{2\pi }\approx \frac{1}{2\pi }\sqrt{\frac{{k}_{1}}{m}}={f}_{R1},\\ {f}_{2}=\frac{{\omega }_{2}}{2\pi }\approx \frac{1}{2\pi }\sqrt{\frac{{k}_{2}}{m}}={f}_{R2}.\end{array}\right..\end{array}\right.$$

By combining Fig. 1c and Eq. (6), it is evident that the mode frequencies of the WCRs closely align with the natural frequency of one of the separate resonators. Assuming that the mode frequencies are consistently dominated by the natural frequency of one resonator, this dominance likely governs the resonant states of the internal resonators, subsequently influencing the energy confinement within the WCRs.

For instance, at the black point (*k*_1_ = *k*_2_ = *k*), both mode frequencies are nearly equal to the natural frequencies of the resonators. As a result, both resonators resonate with equal amplitudes, indicating that energy is evenly distributed between them.

When the stiffness of Resonator 2 decreases (*k*_1_ > *k*_2_) in the yellow region of Fig. 1c, *f*_*R*1_ closely matches the driving frequency (*f*_2_), causing Resonator 1 to resonate with the largest amplitude. In contrast, *f*_*R*2_ deviates from *f*_2_, causing Resonator 2 to gradually lose resonance and its amplitude to decrease. Thus, in the second mode, *f*_2_ of the WCRs is primarily governed by *f*_*R*1_ of the internal resonator.

The AR in the second mode varies linearly when negative perturbations are applied to Resonator 2, as shown in the second mode regions of Fig. 1d. In these regions, the amplitude peaks of Resonator 1 increase, while those of Resonator 2 decrease. Consequently, energy is no longer evenly distributed within the WCRs, but rather confined to Resonator 1. When the stiffness of Resonator 2 increases, the analysis and conclusions follow similarly to the case where the stiffness of Resonator 2 decreases.

The difference between the mode frequencies and natural frequencies leads to distinct resonant states of the resonators within the WCRs. These resonant states determine which resonator vibrates with the largest amplitude and where the energy is confined, offering a qualitative explanation of the energy confinement mechanisms in the WCRs. A subsequent quantitative analysis of the energy confinement, based on the potential energy of the resonators, is then provided.

#### Energy limit of the weakly coupled resonators

During vibration, at the force equilibrium position of the resonator, its velocity is zero, and the amplitude reaches its maximum, corresponding to maximum potential energy and zero kinetic energy. Based on the Equipartition Theorem, the potential energy of the resonator serves as the theoretical basis for estimating its total energy^27^. Therefore, by analyzing the amplitudes of the WCRs in the steady state, a quantitative assessment of the energy distribution within the resonator can be performed. The energy calculation, based on the effective stiffness and displacement of the resonator, is given by:

7 $${E}_{i}=\frac{1}{2}{k}_{i}{X}_{i}^{2}$$

**Fig. 2:**
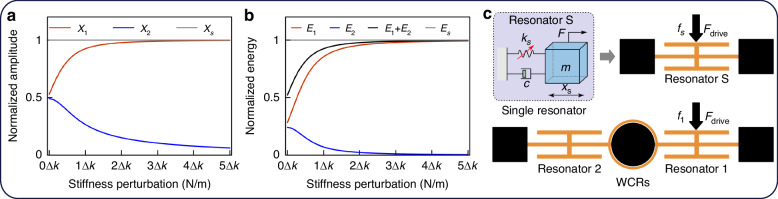
Comparison of amplitudes and energy. **a** Comparison of the normalized amplitudes of WCRs and a single resonator. **b** Comparison of the normalized energy of WCRs and a single resonator. **c** Schematic of a single resonator and WCRs under the identical driving force

As shown in Fig. 2a, the variations in amplitude of the WCRs under stiffness perturbations are illustrated. From Fig. 2a, it can be seen that the amplitude variations of the WCRs are nonlinear. As stiffness perturbations increase, the amplitude of Resonator 1 initially rises rapidly before gradually leveling off, while the amplitude of Resonator 2 first decreases sharply and then slows down.

Using Eq. (7), the energy distribution variation of the WCRs with increasing perturbations is obtained, as shown in Fig. 2b. In this figure when no stiffness perturbation is applied, the displacements of both resonators are almost identical, and their energies are nearly equal. However, as stiffness perturbations increase, the energy of Resonator 1 continues to rise in parallel with its increasing amplitude, while the energy of Resonator 2 gradually decreases as its amplitude diminishes.

Interestingly, by observing the black curve in the figure, it is apparent that the total energy of the WCRs, obtained by summing the energies of the two resonators, is not constant. This indicates that the energy confinement of the WCRs is not merely a spatial redistribution of fixed energy, but rather a dynamic process where the total energy increases as the energy of Resonator 1 grows.

As shown in Fig. 2a, b, both the amplitude and energy variations appear to approach a limit value. Given that the mode frequency of the WCRs is dominated by the natural frequency of the single resonator, it naturally raises the question of whether this energy limit in the WCRs is still related to the single resonator.

A single resonator with the same effective parameters as the resonators inside the WCRs can be constructed, as shown in Fig. 2c. Under the same driving conditions, the driving force *F*_*drive*_ on the single resonator is identical to that of the WCRs. Since the effective parameters of the resonators are the same, the effective stiffness of the WCRs is twice that of the single resonator. Therefore, under the same driving force, the amplitude of the WCRs is half that of the single resonator^31^.

The amplitude and energy comparison between the single resonator and the WCRs, under the same driving force, is shown in Fig. 2a, b. When no stiffness perturbation is applied, the amplitude of the WCRs is approximately half that of the single resonator. However, as the stiffness perturbation increases, the amplitude of Resonator 1 not only increases but also approaches that of the single resonator, as shown in Fig. 2a.

Further comparing the energy distribution, as seen in Fig. 2b, when no stiffness perturbation is applied, the amplitude of the WCRs is half that of the single resonator. Based on the amplitude-square relationship in Eq. (7), the energy of each resonator is approximately one-quarter of that of the single resonator, so the total energy of the WCRs is half of that of the single resonator. As the stiffness perturbation increases, the energy concentrates in Resonator 1. Notably, the energy growth trend of Resonator 1 mirrors the total energy growth of the WCRs, both gradually approaching the total energy of the single resonator. Under the largest stiffness perturbation shown in the figure, the total energy of the WCRs nearly equals that of the single resonator, with Resonator 1 dominating the energy distribution.

Upon quantitatively analyzing the energy confinement in the WCRs, it is evident that there is a close relationship between the WCRs and the single resonator in terms of amplitude and energy. The coupling structure of the WCRs primarily serves to transfer energy between the two resonators, with the amount of energy transferred depending on the effective parameters of the internal resonators. If the effective parameters of the two resonators are equal, the coupling structure effectively transmits energy, allowing both resonators to resonate at the same mode frequency and share the energy equally within the WCRs. However, since the effective stiffness of the two resonators is twice that of the single resonator, their amplitudes and total energy are halved.

If the effective parameters of the two resonators differ significantly, the driving force will only maintain resonance in one resonator, while the other hardly vibrates. In this case, the coupling structure transmits very little energy, and one resonator dominates the energy of the WCRs. As a result, the effective stiffness of the WCRs becomes almost identical to that of the single resonator. During this process of energy confinement, the energy limit of the WCRs is determined by the single resonator with the same effective parameters, and the energy distribution process is governed by the variation in stiffness.

In summary, the phenomenon in which a single resonator predominantly governs the energy confinement in WCRs, driven by the dominance of the natural frequencies of the internal resonators over the mode frequencies of the WCRs, is referred to as the “modal dominance” of WCRs.

### Virtually coupled resonators based on the modal dominance

The frequency shifts between the mode frequencies and the natural frequencies lead to energy confinement within WCRs, and a similar effect can be achieved in two separate resonators. Unlike WCRs, which rely on a physical coupling structure for energy confinement, VCRs achieve this effect through frequency offsets. Specifically, a frequency offset is introduced between two discrete resonators, where the frequency of one resonator (Resonator 1) drives the other (Resonator 2). Although there is no physical coupling, the interaction through frequency alignment acts as a virtual coupling mechanism. This interaction effectively replicates the energy confinement and AR output characteristics of WCRs. This design innovation preserves the sensitivity and performance advantages of WCRs while offering structural simplicity and enabling new control schemes.

**Fig. 3:**
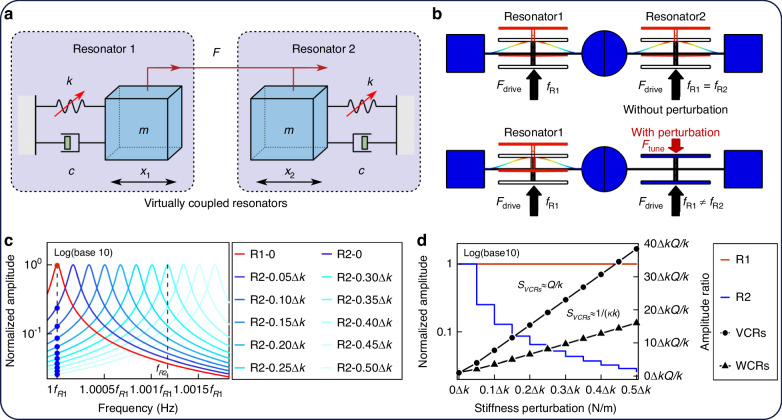
Models, amplitudes, and AR variations of VCRs. **a** Mass–stiffness–damper model of the VCRs. **b** COMSOL simulation of VCRs with and without perturbation. **c** Amplitude–frequency responses of Resonator 1 and Resonator 2 under stiffness perturbations applied to Resonator 2. **d** Amplitudes and AR of VCRs compared to WCRs

In Fig. 3a, both resonators are driven simultaneously by the same force, with the driving force frequency set to *f*_*R*1_. The amplitudes of the two resonators (*X*_*Ri*_) in the separate resonators are expressed as follows:

8 $$\left\{\begin{array}{c}{X}_{R1}\left({\omega }_{R1}\right)=\frac{1}{-m{\omega }_{R1}^{2}+{jc}{\omega }_{R1}+{k}_{1}}{F}_{1}\left({\omega }_{R1}\right),\\ {X}_{R2}\left({\omega }_{R1}\right)=\frac{1}{-m{\omega }_{R1}^{2}+{jc}{\omega }_{R1}+{k}_{2}}{F}_{1}\left({\omega }_{R1}\right).\end{array}\right.$$

When the stiffness of Resonator 2 is altered, the amplitudes of both resonators at *f*_*R*1_ are recorded, and the amplitude–frequency responses are shown in Fig. 3c. Theoretically, the amplitude–frequency response of Resonator 1 remains unchanged, as no stiffness perturbation is applied to it. In contrast, the amplitude–frequency response of Resonator 2 shifts to the right due to the positive stiffness perturbation. As a result, the amplitude of Resonator 1 at *f*_*R*1_ stays constant, while the amplitude of Resonator 2 decreases, since *f*_*R*2_ moves away from *f*_*R*1_, similar to the frequency shifts observed in WCRs. The output of the separate resonators is represented by the AR_*VCR*_ between the two resonators at *f*_*R*1_. The AR_*VCR*_ at *f*_*R*1_ and its sensitivity can be expressed as follows:

9 $$\left\{\begin{array}{ll}{\mathrm{AR}}_{VCRs}=\frac{X_{R1}\left({\omega}_{R1} \right)}{X_{R2}\left( {\omega}_{R1} \right)}=\frac{-m{\omega}_{R1}^{2}+jc{\omega}_{R1}+k+\varDelta k}{-m{\omega}_{R1}^{2}+jc{\omega}_{R1}+k},\\ \left|{S}_{AR-VCRs} \right|=\frac{Q}{k}.\end{array}\right.$$

According to the Eqs. (8) and (9), the amplitudes of the two separated resonators and their AR are shown in Fig. 3d. The AR of the two resonators at *f*_*R*1_ demonstrates excellent linearity with respect to stiffness perturbations, highlighting its potential for sensing input variations. Unlike WCRs, there is no substantial coupling structure between the two separated resonators; they are only connected by the same driving force. The mechanism of amplitude changes and AR output, originating from frequency shifts, is derived from WCRs. Consequently, resonators that utilize the AR output from the frequency shifts of two separate resonators are referred to as VCRs.

The sensitivity of the AR_*VCRs*_ is proportional to the quality factor *Q*, as indicated in Eq. (9). In contrast, the sensitivity of WCRs is proportional to 1/*κ*. Given the condition for avoiding mode aliasing, the sensitivity of VCRs is inherently higher than that of WCRs.

### The improvement of amplitude ratio bandwidth

**Fig. 4:**
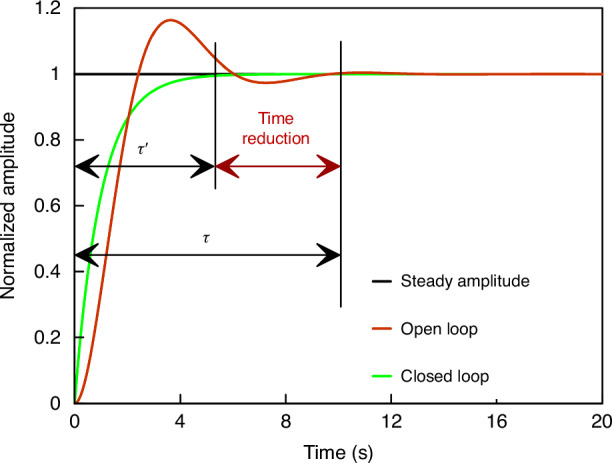
Schematic of reduction in settling time with and without a closed-loop control for resonators

Resonator amplitudes take a considerable amount of time to stabilize at a new steady-state value in a high-vacuum environment^32,33^. With proportional-integral (PI) control in the closed-loop circuit for resonators, the faster electronic settling time of the circuit compensates for the slower mechanical stabilization of the resonator amplitudes in high vacuum, as shown in Fig. 4.

**Fig. 5:**
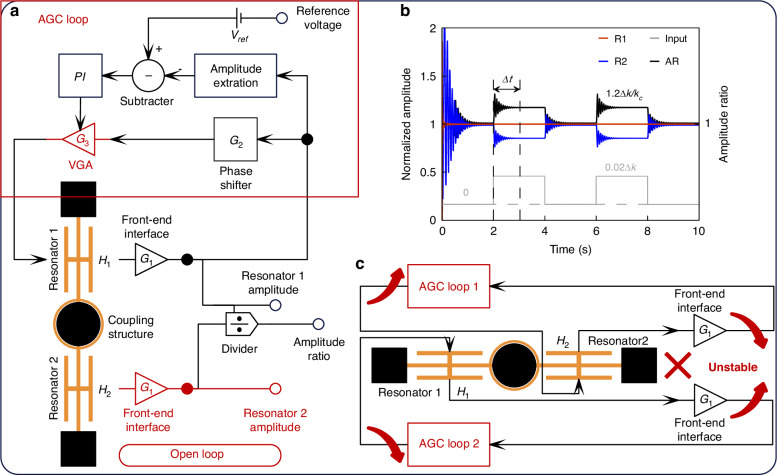
Closed-loop control and AR bandwidth simulation of WCRs. **a** Schematic of closed-loop control with AGC. **b** Simulation of AR under square wave stiffness perturbation input. **c** Schematic of double-closed-loop control for WCRs

Closed-loop control based on automatic gain control (AGC) is widely used in resonators, as shown in Fig. 5a. The AGC loop consists of resonators, a front-end interface, a phase shifter, a control loop, and a variable gain amplifier (VGA). The control loop primarily includes an amplitude extraction circuit, a reference voltage (*V*_*ref*_), a PI controller, and the VGA. The front-end interface amplifies the currents from the resonators, while charge amplifiers (CAs) both amplify and integrate the resonator output currents into voltages that reflect displacement. However, CAs introduce a 90° phase shift in the loop, necessitating a phase shifter to compensate for this shift and satisfy the phase condition required for oscillation^34^. The amplitude extraction circuit processes the output from the front-end interface to produce a DC voltage, which is then compared with *V*_*ref*_ to generate a difference signal that is fed into the PI controller. The PI controller outputs a DC regulation voltage. This voltage is combined with the resonator voltage via the VGA to generate the driving voltage, which is then applied to the resonator’s driving electrode. AGC, incorporating the PI controller and VGA, ensures that the oscillating amplitude matches *V*_*ref*_, maintaining stable oscillations and ensuring system stability.

However, in WCRs, closed-loop control typically regulates only one resonator, leaving the other in an open-loop configuration, as illustrated in Fig. 5a. As a result, the AR of the WCRs settles only when the open-loop resonator reaches stability, leading to a low AR bandwidth, as shown in Fig. 5b. Achieving simultaneous control of both resonators in WCRs could significantly improve bandwidth, but directly implementing two closed loops presents challenges. The amplitude variations and gain adjustments in each loop become contradictory, preventing the stable operation of the two resonators. This instability arises because the coupling structure in WCRs causes the amplitudes of the resonators to be mutually influenced by both driving forces under double-resonator-driven conditions. As the amplitudes of the resonators increase or decrease, the two driving forces adjust in opposite directions, as shown in Fig. 5c. As a result, the amplitudes of the resonators fail to reach stable states.

**Fig. 6:**
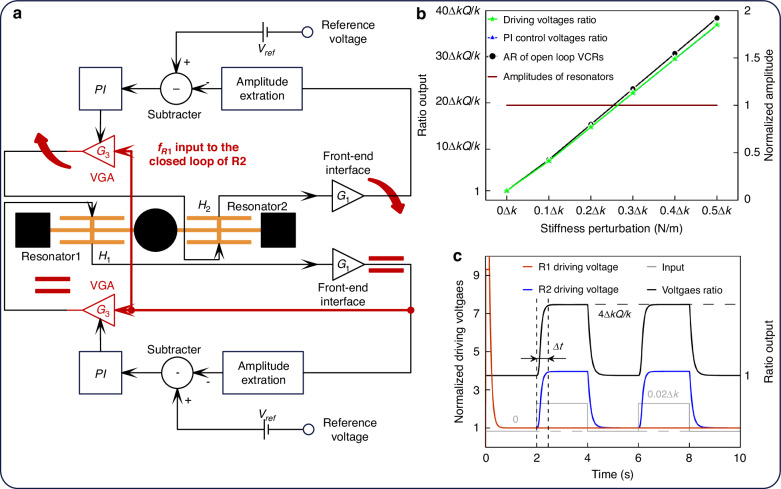
Double-closed-loop control and AR bandwidth simulation of VCRs. **a** Schematic of double-closed-loop control with two AGC loops. **b** Simulation of AR in open-loop and the ratio of driving voltages and PI control voltages in the closed loop of VCRs. **c** Simulation of AR under square wave stiffness perturbation input

VCRs, in contrast, do not require a coupling structure and can achieve a similar AR output, making it possible to improve bandwidth without compromising AR sensitivity. The double-closed-loop schematic for VCRs is shown in Fig. 6a, featuring two identical closed loops to control the two resonators. The key distinction from the double-closed-loop schematic of WCRs in Fig. 5c is that the frequency of Resonator 2 is derived from the loop of Resonator 1, ensuring that the AR output reflects the amplitudes of both resonators at *f*_*R*1_. In this double-closed-loop schematic for VCRs, the amplitudes of both resonators remain constant, regulated by their respective reference voltages due to the AGC method. As a result, the AR output is determined by the ratio of the driving voltages or PI control voltages. Simulation results demonstrate that the AR of VCRs in open-loop mode closely aligns with the ratio between the driving voltages or PI control voltages, as shown in Fig. 6b. This validates the feasibility of the double-closed-loop schematic for VCRs. By simultaneously controlling both resonators, the bandwidth of the AR can be enhanced, as illustrated in Fig. 6c.

## Materials and methods

WCRs and VCRs with identical resonator parameters were designed to demonstrate the enhanced AR sensitivity and bandwidth of VCRs. The resonator parameters are summarized in Table 1. The natural frequency of the resonators in the coupled system is simulated as ~21,433 Hz, while the two resonant modes in the WCRs showed simulated frequencies of 20,687 Hz and 20,703 Hz, respectively. The frequency difference between these two modes is approximately 16 Hz. Based on the theoretical frequency difference and mode frequencies of the WCRs, the calculated coupling coefficient is ~7.88 × 10^−4^. The coupling stiffness of the annular coupler is estimated as 0.118 N/m^22^.

**Table 1:** Parameters of resonators

Parameters	Value
Resonator beam length	1300 μm
Resonator beam width	15 μm
Resonator beam thickness	50 μm
Annular coupler width	6 μm
Resonator Effective mass	8.79 × 10^−9^ kg
Resonator Effective stiffness	149.3 N/m
Resonator natural frequency	21,433 Hz
First mode frequency of WCRs	20,687 Hz
Second mode frequency of WCRs	20,703 Hz

**Fig. 7:**
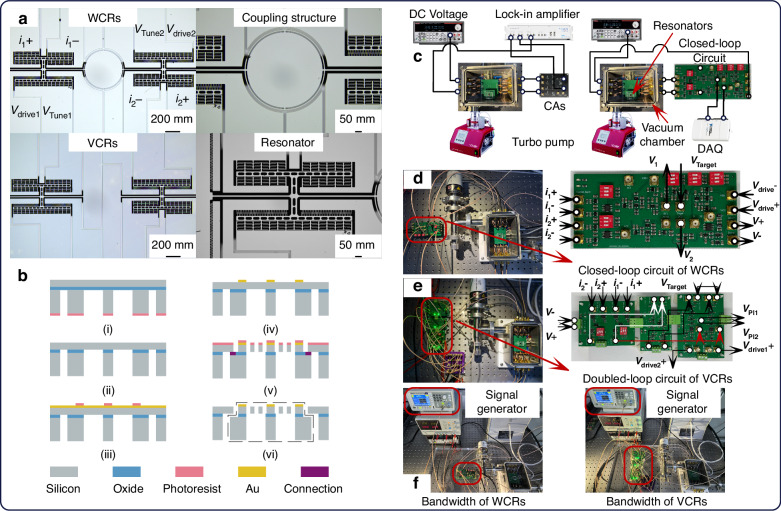
The chip under test and the experimental setup. **a** Images of the coupled resonators. **b** Fabrication process for the coupled resonators. **c** Schematic of open-loop and closed-loop test. **d** Closed-loop test of WCRs. **e** Closed-loop test of VCRs. **f** Bandwidth test of WCRs and VCRs

Images of the WCRs, their coupling structure, VCRs, and resonators are shown in Fig. 7a. The central component of the WCRs includes the annular coupler^35^, while the middle section of the VCRs consists of two separate anchors that connect to the isolated resonators within the VCRs.

These structures are fabricated using a dicing-free Silicon-on-Insulator (SOI) process, with a 50 µm thick device layer^36,37^. The fabrication process, illustrated in Fig. 7b, involves the following main steps: (i) deposit photoresist, pattern the substrate layer features, and etch them using deep reactive ion etching (DRIE) until reaching the buried oxide layer; (ii) remove the photoresist and release the exposed oxide from the backside of the SOI wafer using a 49% hydrofluoric acid (HF) solution; (iii) sputter a 200 nm gold layer onto the device layer, deposit photoresist, and pattern; (iv) create contact pads, then remove the photoresist; (v) deposit photoresist, pattern the device layer, and etch features using DRIE to form the resonator structures. At this stage, the WCRs remain mechanically connected to the wafer via the connection area; (vi) remove the photoresist and separate the WCRs from the wafer by releasing the connection area.

The WCRs and VCRs are tested in a vacuum chamber maintained by a turbo pump, ensuring the necessary vacuum environment. DC voltage sources supply the bias and tuning voltages required for the resonators. For the open-loop test, CAs and a lock-in amplifier are used, while the closed-loop test employs a dedicated closed-loop circuit and a data acquisition board (DAQ), as shown in Fig. 7c.

Figure 7d, e displays the closed-loop circuit setups for the WCRs and VCRs. Both circuits include four current inputs for two sets of differential currents (*i*_1+_, *i*_1-_, *i*_2+_, and *i*_2-_), reference voltages (*V*_*Target*_), and two power supply ports (*V*+ and *V*−). These closed-loop circuits are equipped with interfaces for the amplitude voltages corresponding to the resonators and the loop feedback driving voltages, allowing these signals to be observed and recorded during the experiment.

The key difference between the two circuits lies in the double-closed-loop configuration for the VCRs. This setup incorporates two identical AGC loops, with the amplitude voltage of Resonator 1 serving as an input to the closed loop of Resonator 2. This ensures that Resonator 2 vibrates at the frequency *f*_*R*1_, enabling synchronized operation and improved performance.

The bandwidth test for WCRs and VCRs involves using a signal generator to supply a square wave voltage with varying frequencies to the resonators. The ARs of WCRs and VCRs are then measured and recorded from the closed-loop circuits, as illustrated in Fig. 7f.

**Fig. 8:**
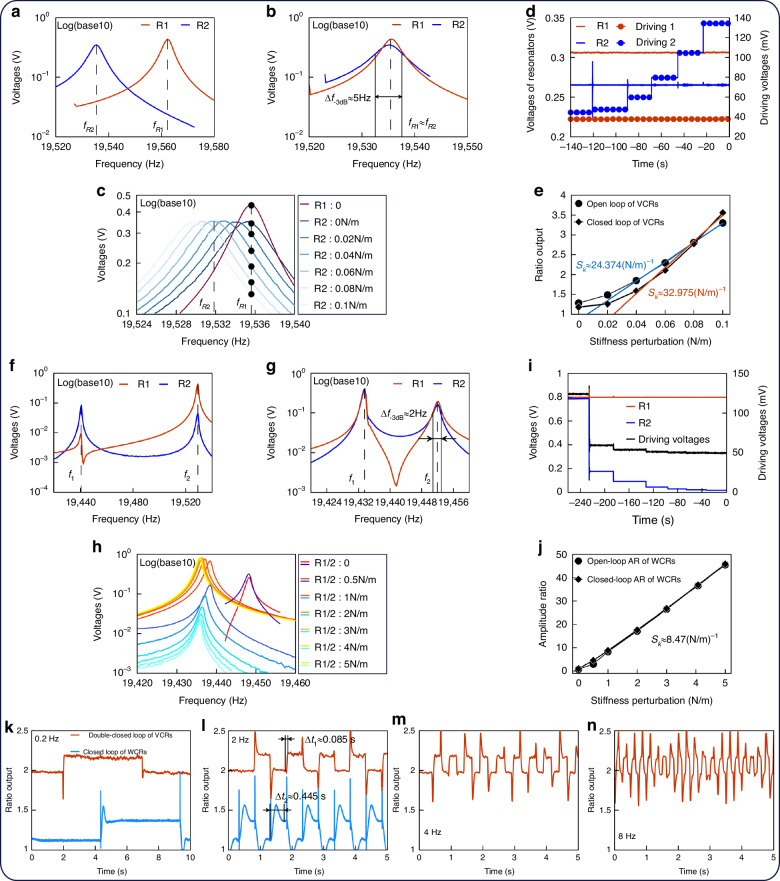
Test results of WCRs and VCRs. **a** Open-loop amplitude–frequency response of VCRs before tuning. **b** Open-loop amplitude–frequency response of VCRs after tuning. **c** Open-loop amplitude–frequency response of VCRs with input. **d** Double-closed-loop response of VCRs. **e** Comparison of open-loop and closed-loop AR outputs for VCRs. **f** Open-loop amplitude–frequency response of WCRs before tuning. **g** Open-loop amplitude–frequency response of WCRs after tuning. **h** Open-loop amplitude–frequency response of WCRs with input. **i** Closed-loop response of WCRs. **j** Comparison of open-loop and closed-loop AR outputs for WCRs. **k**–**n** Bandwidth comparison between WCRs and VCRs with square wave voltage inputs at **k** 0.2 Hz, **l** 2 Hz, **m** 4 Hz, and **n** 8 Hz

The VCRs are driven using a 50 mV swept voltage, with a 5 V DC bias voltage applied. Both resonators in the VCRs are set with tuning voltages of 5 V to ensure that no stiffness adjustment occurs via the tuning electrodes. The amplitude–frequency responses of the VCRs are shown in Fig. 8a, with natural frequencies (*f*_*R*1_ and *f*_*R*2_) of 19.562 kHz and 19.535 kHz, respectively. Due to fabrication tolerances, variations in stiffness result in slightly different natural frequencies for the two resonators, with Resonator 1 having greater stiffness than Resonator 2.

When a 9.2 V tuning voltage is applied to Resonator 1, the natural frequencies of the two resonators become nearly identical, as shown in Fig. 8b. After this adjustment, the resonance frequency of both resonators is ~19.535 kHz in a vacuum environment of 0.012 Pa. Using the resonant peak corresponding to *f*_*R*2_ in Fig. 8b and the −3 dB bandwidth frequency Δ*f*_−3dB_ ≈ 5 Hz, the *Q* of the resonator is estimated as *Q* = *f*_*R*2_/Δ*f*_−3dB_ ≈ 3907.

Starting from the initial tuning voltage applied to Resonator 1, the tuning voltage on Resonator 2 is gradually increased to reduce its effective stiffness, simulating the input. The tuning voltages applied to Resonator 2 and the corresponding effective stiffness values are summarized in Table 2. For different tuning voltages, the amplitude–frequency response curves of both resonators are recorded, with the amplitude values of each resonator noted at *f*_*R*1_. Within a stiffness input range of 0 to 0.1 N/m, the amplitude–frequency responses of VCRs under varying stiffness perturbations are shown in Fig. 8c. At *f*_*R*1_, the maximum amplitude of Resonator 1 remains constant, while the amplitude of Resonator 2 progressively decreases as the stiffness perturbation increases.

**Table 2:** Tuning voltages on Resonator 2 of VCRs

Tuning voltages (V)	Effective stiffness (N/m)
5.000	0.00
5.877	0.02
6.240	0.04
6.518	0.06
6.753	0.08
6.960	0.10

Using the same tuning voltages and effective stiffness perturbations as listed in Table 2, the variations in amplitude and driving voltages of the VCRs under double-closed-loop control are shown in Fig. 8d. The reference voltages for both resonators are set to 300 mV. Within the AGC loop, the amplitude voltages of both resonators remain stable. The driving voltage of Resonator 1 remains constant, while the driving voltage of Resonator 2 continuously increases. Under double-closed-loop control, the AR of the VCRs is derived by dividing the driving voltages. A comparison between the open-loop AR and the driving voltage ratio of the VCRs is presented in Fig. 8e.

Using the *Q* of the VCRs and the initial stiffness of the resonators, the theoretical AR sensitivity is estimated to be approximately 26.2 (N/m)^−^^1^, based on Eq. (9). As shown in Fig. 8e, the open-loop sensitivity of the VCRs is about 24.4 (N/m)^−1^, while the closed-loop sensitivity, derived from the driving voltage ratio, is approximately 33.0 (N/m)^−1^. The open-loop sensitivity closely aligns with the theoretical value, whereas the closed-loop sensitivity is higher. This discrepancy may arise from the multiple stages in the analog circuit. Excess gain in the closed loop could cause the measured driving voltage of Resonator 2 to be larger, or potentially the driving voltage of Resonator 1 to be smaller. Despite this, the results confirm the feasibility of the AR output of VCRs and validate its double-closed-loop control mechanism.

Similarly, the open- and closed-loop test results for WCRs are presented in Fig. 8f–j. The WCRs were driven with a 50 mV swept voltage and a 5 V DC bias voltage. The amplitude–frequency responses of the WCRs are depicted in Fig. 8f, with *f*_1_ and *f*_2_ at 19.441 kHz and 19.529 kHz, respectively. Resonator 1 exhibits greater stiffness than Resonator 2, resulting in a frequency difference of 88 Hz. When an 11.8 V tuning voltage is applied to Resonator 1, the amplitudes of both resonators become nearly identical, as shown in Fig. 8g. After this adjustment, the mode frequencies of the WCRs are ~19.433 kHz and 19.452 kHz, with a frequency difference of approximately 19 Hz. The vacuum level during the experiment is ~0.022 Pa. The tuning voltages applied to Resonator 2 and the corresponding effective stiffness values for the WCRs are summarized in Table 3. Under a stiffness input range of 0 to 5 N/m, the amplitude–frequency responses of the WCRs are depicted in Fig. 8h. The amplitude voltage of Resonator 1 increases, while that of Resonator 2 decreases. The closed-loop test results for the WCRs are shown in Fig. 8i, where the reference voltage for Resonator 1 is set to 800 mV. Within the AGC loop, the amplitude voltage of Resonator 1 remains constant, the driving voltage of Resonator 1 continuously decreases, and the amplitude voltage of Resonator 2 steadily declines. A comparison of the open-loop and closed-loop ARs of the WCRs is illustrated in Fig. 8j.

**Table 3:** Tuning voltages on Resonator 2 of WCRs

Tuning voltages (V)	Effective stiffness (N/m)
5.000	0.0
9.384	0.5
11.199	1.0
13.767	2.0
15.738	3.0
17.399	4.0
18.862	5.0

The ARs obtained from both the open-loop and AGC closed-loop tests are largely consistent, demonstrating a stiffness sensitivity of ~9.03 (N/m)^−1^. Based on the coupling stiffness of the WCRs, the theoretical stiffness sensitivity is estimated to be about 8.47 (N/m)^−^^1^, as derived from Eq. (2). The experimentally measured sensitivity aligns closely with the theoretical value, with no significant deviation observed.

By comparing the stiffness sensitivities of the VCRs and WCRs, as shown in Fig. 8e and j, the open-loop sensitivity of the VCRs is ~2.7 times greater than that of the WCRs. Experimentally, the *Q* of the VCRs is around 3907, while the coupling coefficient *κ* of the WCRs is ~7.88 × 10^−4^. The product of these two values is *κQ* ≈ 3.08, satisfying the condition of avoiding mode aliasing. Since the AR sensitivity of the VCRs is proportional to *Q*, and the AR sensitivity of the WCRs is proportional to 1/*κ*, the theoretical AR sensitivity of the VCRs is predicted to be approximately three times that of the WCRs. This prediction aligns well with the experimental results, showing minimal deviation from the theoretical estimation.

When input voltage signals from the signal generator have frequencies of 0.2 Hz, 2 Hz, 4 Hz, and 8 Hz, the response curves of the AR under single-closed-loop control of the WCRs and double-closed-loop control of the VCRs are presented in Fig. 8k–n. At input signal frequencies below 1 Hz, both control schemes can respond in real time, as shown in Fig. 8k. However, as the input signal frequency increases, for instance, with a bandwidth of 2 Hz, the stabilization time for the open-loop resonator in the single-closed-loop control scheme becomes insufficient. This results in the AR output reaching a critical response rate and failing to provide stable outputs for faster-varying signals, as illustrated in Fig. 8l. Conversely, under double-closed-loop control, the AR output remains effective even at an input signal bandwidth of 8 Hz, as depicted in Fig. 8m, n.

According to Fig. 8l, the settling times of the double-closed-loop control of the VCRs and the single-closed-loop control of the WCRs are Δ*t*_1_ ≈ 0.085 s and Δ*t*_2_ ≈ 0.445 s, respectively. Based on the estimated settling times, the bandwidths of the VCRs and WCRs in the experiment are about 12 Hz and 2.25 Hz, respectively.

## Conclusion

This study has explored the energy confinement within WCRs and introduced the innovative concept of VCRs, which simplifies the structural requirements of traditional mode-localized sensors while maintaining, and in some aspects enhancing, their performance capabilities. Our exploration of modal dominance demonstrates that the mode frequencies in WCRs are predominantly governed by the natural frequencies of the internal resonators. This discovery not only enriches our understanding of energy confinement in microsystems but also lays the groundwork for more advanced sensor designs.

By eliminating the physical coupling structures and utilizing frequency offsets to couple two discrete resonators, VCRs achieve similar energy confinement and AR outputs as traditional physically coupled counterparts but with improved sensitivity and bandwidth. The double-closed-loop control scheme developed for VCRs further optimizes performance, addressing one of the fundamental challenges in the field—balancing sensitivity with operational bandwidth. Experimental validations affirm that VCRs can achieve a 2.7-fold improvement in AR sensitivity and at least a four-fold increase in bandwidth compared to WCRs under similar operational conditions.

Future work will concentrate on refining the structural design of VCRs and advancing the digital double-closed-loop control scheme to extend bandwidth capabilities further. Despite the isolation of resonators within the device layer in the fabricated VCRs, vibrations from the base layer may still induce minor coupling effects between the resonators under substantial driving forces. Efforts will continue to refine the structural design to enhance isolation of the resonators and minimize these undesired effects. Additionally, simulation and experimental results indicate that the double-closed-loop control scheme significantly reduces stabilization time for open-loop resonators, thus enhancing AR output bandwidth. With the implementation of an advanced digital double-closed-loop control scheme and further optimization of electromechanical parameters, the bandwidth could potentially extend to tens of hertz. This advancement holds significant promise for overcoming the traditional bandwidth limitations associated with mode-localized sensors.
